# Do lifetime contest costs affect the evolution of assessment strategies? A meta‐analysis

**DOI:** 10.1111/1365-2656.70058

**Published:** 2025-05-26

**Authors:** Clara Massote, Alexandre V. Palaoro, Gareth Arnott, Domhnall Jennings, Paulo Enrique Cardoso Peixoto

**Affiliations:** ^1^ Departamento de Ecologia, Conservação e Manejo da Vida Silvestre Universidade Federal de Minas Gerais Belo Horizonte Minas Gerais Brazil; ^2^ Departamento de Zoologia Universidade Federal do Paraná Curitiba Paraná Brazil; ^3^ School of Biological Sciences Queen's University Belfast Belfast UK

**Keywords:** agonistic interactions, animal contests, assessment rules, decision rules, evolutionary game‐theory, mutual assessment, self‐assessment

## Abstract

1. In animal contests, the decision to flee or to remain in a contest is crucial to the individual's fitness because a wrong decision can dramatically change the costs individuals accrue over their lifetime.
2. Empirical evaluations of the rules that individuals adopt to remain on a contest often provide support for two major possibilities: self‐assessment strategies, in which each individual remain in the contest until reaching its own cost threshold, and a mutual‐assessment strategy, in which individuals assess the rival's capacity to sustain or to impose costs during the contest and decide to flee when they consider that the rival is stronger.
3. However, it is unclear what drives the evolution of different assessment strategies among species. A factor that may be key to understanding such variation is contest cost. If contests provide a fast and/or large cost accrual, mutual assessment strategies may be favoured because they avoid contests in which individuals always reach their own cost threshold. On the other hand, in species in which cost accrual during the contest is slow and/or small, self‐assessment may prevail.
4. Here, we performed a meta‐analysis using information on the way individuals engage in contests in different species. Our goal is to test the hypothesis that contests involving high‐cost accrual, such as contests in species in which rivals use weapons, can favour the evolution of mutual assessment strategies, while contests with lower costs, such as contests without physical contact, should favour the evolution of self‐assessment strategies.
5. We obtained 80 effect sizes spanning 36 species. Contrary to our hypothesis, we found that species with high‐cost contests consistently adopted self‐assessment strategies, while species with low‐cost contests did not present a consistent assessment strategy.
6. We suggest that high‐cost contests may evolve in species in which individuals experiencing defeat or opting to flee from a contest have a significant decrease in their reproductive success. Consequently, individuals would be compelled to engage in contests regardless of their opponent. In low‐cost contests, however, we suggest that any potential selective pressure for a specific assessment strategy is relaxed, which could explain the diversity of assessment strategies observed in this category.

## Graphical abstract

Our meta‐analysis reveals unexpected patterns in the evolution of assessment strategies during animal contests. We show that self‐assessment dominates in high‐cost fights, challenging existing theories. This study provides novel insights into the link between fighting costs and behavioural evolution, with implications for understanding fitness consequences across diverse species.

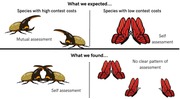

## 1 INTRODUCTION

When individuals compete for resources, the decision to engage in an agonistic contest or to flee from it can significantly impact fitness, as this decision will influence the costs and the benefits the individual might accrue (Hardy & Briffa, 2013). Fleeing from a contest means accepting the costs already incurred and that the disputed resource will be lost. Conversely, staying in the contest means accruing additional energetic and time costs, including the possibility of suffering injury while still losing the disputed resource (Hardy & Briffa, 2013). If the individual flees, the timing of the decision to leave early or late can result in a substantial disparity in costs, particularly when contests involve the use of costly behaviours that increase the likelihood of injury or exhaustion (Archer, 1988; Guo & Dukas, 2020; Huntingford, 2013; Palaoro & Peixoto, 2022). Thus, a suboptimal decision to remain in a contest can have lifelong fitness consequences in terms of survival and reproduction. Given the close relationship between decision‐making during contests and fitness (Arnott & Elwood, 2008; Guo & Dukas, 2020), it is unsurprising that these decisions are subject to multiple selective pressures.

Several evolutionary game‐theory models seek to explain how individuals make decisions during contests (reviewed by Arnott & Elwood, 2009). In general, such models may be divided into two major groups: self‐ and mutual assessment models (Chapin et al., 2019; Taylor & Elwood, 2003). According to self‐assessment models, individuals flee when they reach a cost threshold during the contest, with this threshold being dependent on the individual's own capacity to accumulate costs during the contest (Parker, 1974). As such, weaker individuals will reach their cost threshold first and give up. Self‐assessment models can be further subdivided regarding how costs can be accrued. The war of attrition (WOA) models, including the ‘war of attrition without assessment’ (WOA‐WA, Mesterton‐Gibbons et al., 1996) and ‘energetic war of attrition’ (E‐WOA, Payne & Pagel, 1997), posit that cost accrual depends solely on the individual's actions during the contest; the ‘cumulative assessment model’ (CAM, Payne, 1998) incorporates opponent's actions into cost accrual. Such actions in CAM are often represented by physical aggression performed to increase costs, thereby, forcing an opponent to reach its cost threshold first. Therefore, despite differences in the assumptions of how individuals might incur costs during a contest, WOA, E‐WOA and CAM have the same prediction that an individual will give up only after reaching its own cost threshold. In contrast to self‐assessment, mutual assessment models assume that opponents assess the relative capacity of the opponent to sustain or impose costs during the contest (collectively called Resource Holding Potential or RHP—Parker, 1974), thought to involve comparisons of their own RHP against the opponent's RHP (Elwood & Arnott, 2012). This strategy is encompassed in the ‘sequential assessment model’ (SAM, Enquist & Leimar, 1983), in which each rival compares their RHP with their opponent's, giving up when it considers itself to be the weaker of the pair. The selective advantage of mutual assessment is that contestants can avoid engaging in escalated contests that they would likely lose, whereas in self‐assessment models, contestants should always remain in the contest until the weaker rival reaches its cost threshold. It is important to note that for all models (WOA, E‐ WOA, CAM and SAM), costs are a central consideration in decision‐making with the difference related to how and, ultimately, the amount of costs accrued. Given the central part that costs play in these models, mutual assessment has frequently been suggested as the ‘optimal’ solution to avoid accruing the ‘unnecessary costs’ of continuing in a contest when the individual is clearly the weaker of the pair (Arnott & Elwood, 2009).

A major puzzle with empirical investigations of the assessment strategies adopted by rivals is the wide variation observed among species (Pinto & Peixoto, 2019). Such variation may indicate that some overlooked factor may explain why some species tend to adopt self‐assessment, while others adopt mutual assessment strategies to decide when to flee. An untested possibility is that the differences in how contest costs are accrued over the lifetime of the individuals may represent a key factor to explain why different species provide distinct support to each assessment model. During contests, individuals may accumulate immediate costs such as energy depletion and injuries. However, such costs can accumulate through different interactions, which can then incur lifetime costs and further impact the individual's fitness. For example, in the fruit fly *Drosophila melanogaster*, males that engage in contests more frequently have lower survivorship than males that engage less frequently (e.g. Guo & Dukas, 2020). Under this framework, self‐assessment and mutual assessment strategies will have different predictions regarding how much cost individuals accrue throughout their lifetime. Contests in species in which individuals adopt self‐assessment will last until the loser reaches its cost threshold. This is true even for individuals that make contest decisions according to CAM, because although injuries caused by opponents affect the rate of cost accrual, each individual should give up when reaching its own cost threshold. On the other hand, in species in which the individuals have the same cost threshold but adopt mutual assessment, contests between rivals with similar RHPs will escalate and reach high costs, whereas those between rivals that differ in RHP can be quickly resolved before reaching a maximum cost threshold (Lane & Briffa, 2017). Since an individual is expected to engage in multiple contests during its lifetime, it is expected that it will be the weaker in a proportion of those contests if individuals find rivals randomly. But even if individuals can choose weaker opponents, an individual will be chosen by a stronger rival in some contests. Thus, if the rate of cost accrual during the contest is equal among species, the lifetime costs accrued by individuals in species that adopt self‐assessment should be higher than those that adopt mutual assessment, since losers adopting self‐assessment will always reach their cost threshold.

It is highly unlikely, however, that the rate of cost accrual during contests is similar among species. For example, there are species in which individuals use weapons that can injure the rival during the contest (e.g. Banks Jr et al., 1968; McCullough & Emlen, 2013), while in other species, contests are based on non‐contact displays that primarily involve energy depletion (e.g. Kemp, 2013). Such variation in the costs accrued during contests may affect when self‐ or mutual assessment strategies should be favoured since assessment strategies that provide a greater lifetime cost accumulation should be selected against in species that show high‐cost contests.

The best way to investigate contest costs would involve identifying the lifetime consequences of losing each contest. However, such information is unknown for most species. Therefore, indirect estimations are necessary if we want to investigate whether contest costs might affect the evolution of assessment strategies. Behavioural and morphological traits that impact the capacity to impose costs on rivals during contests represent suitable estimations. For instance, contests of the mantis shrimp *Neogonodactylus bredinii* consist of visual displays, the exchange of high‐force strikes, and the use of raptorial appendages, which can impose the cost of an injury (Caldwell, 1987; Green & Patek, 2018). If individuals of this species adopt self‐assessment during contests, losers could accumulate a large amount of injuries that could negatively impact their survival and compromise the opportunity for future reproductions after a few contests. There is also the possibility that species with highly exaggerated weapons use them mostly for displays, reducing the potential costs accrued during contests. But the energetic costs of maintaining and displaying an exaggerated structure should be high. For example, in the fiddler crab *Austruca pugilator*, the enlarged claw of males (used for both display and physical contact during the contest) has a strong effect on male resting metabolic rates (Allent & Levinton, 2007). Under these scenarios (i.e. species with weapons used during contests), a mutual‐assessment strategy should be favoured and, in fact, has been found (Green & Patek, 2018). On the other hand, there are species in which the cost accrual is smaller. For instance, in the butterfly *Eumaeus toxea*, individuals do not present weaponry, and the contests occur without physical contact. The main cost in *E. toxea* is the energy spent in aerial displays during the contest (Martínez‐Lendech et al., 2007). Due to the comparative low rate of cost accrual, a self‐assessment strategy should not increase the accumulated cost to a point that reduces the individual's lifetime survival. Therefore, it may be that the rate of cost accrual during the contest affects when self or mutual assessment should be favoured. However, as far as we know, there has never been a comparison between contest costs and assessment strategy across species.

To perform a cross‐species comparison regarding the effect of cost accrual during contests on the evolution of assessment strategies, it is necessary to investigate species that belong to distantly related groups and have distinct contest behaviours. To reach this goal, we performed a meta‐analysis comparing the assessment strategies adopted by species across animals and the potential costs associated with their contests. We hypothesized that in species with cost contests, mutual assessment should be favoured, whereas species with low‐cost contests self‐assessment should prevail.

## 2 METHODS

We followed the PRISMA protocol adapted to ecology and evolutionary studies as much as possible to develop this meta‐analysis (Figure S1; O'Dea et al., 2021).

### 2.1 Eligibility criteria

Our procedure of data acquisition consisted of a two‐step process. First, we needed to gather information that allowed the estimation of the type of assessment adopted by individuals of each species. Second, we needed to estimate contest costs in a way that was comparable among species. For the first step, a standard way to investigate assessment strategies adopted by rivals consists of regressing a measure of contest costs accrual (often contest duration) against individual traits that represent the RHP of losers and winners (Taylor & Elwood, 2003). For this reason, only studies that provided relationships between contest duration and morphophysiological characteristics that indicated the RHP of both contestants, the winner and the loser, were eligible for our meta‐analysis. This relationship could involve rivals in contests under natural or laboratory conditions. We excluded studies where rivals were paired before contests according to a similar morphophysiological characteristic because, in these situations, we would be unable to detect that the trait is a determinant of the contest outcome. For the second step, we included studies that provided information on the contest behaviour adopted by individuals of each species. If an article included in the first step did not provide such information, we included it as well and searched for information on contest behaviour in additional articles of the same species.

### 2.2 Finding studies

We used the database provided in Pinto et al. (2019) that covered studies published between 2004 and 2019. This contained the necessary information about the correlation between contest duration and contestant morphophysiological characteristics related to contest outcome. We then extracted the size effects reported in the original study as traits correlated to contest duration. We conducted an additional search in Scopus and Web of Science databases from 1 January 2019 until 1 October 2021 using the same key words as Pinto et al. (2019): ‘animal contests’, ‘RHP’, ‘resource holding potential’, ‘contest resolution’, ‘SAM’, ‘sequential assessment model’, ‘WOA’, ‘war of attrition’, ‘E‐WOA’, ‘energetic war of attrition’, ‘CAM’, ‘cumulative assessment model’ and ‘contest duration’. All keywords were used with the “OR” Boolean operator in a single search in each database.

### 2.3 Study selection and data collection process

Despite the use of keywords specific to animal contests, our initial search resulted in many studies unrelated to contest behaviour. Therefore, we first inspected the title of each study and excluded those that were clearly unrelated to animal contests (*N* = 1155). Among the remaining studies (*N* = 49), we read the text to search for correlations between contest duration and loser and winner traits that indicated RHP. We selected studies that provided, directly or indirectly, this information. To control for possible dependencies in our data (see how we used them in the statistical analysis section), alongside the main variables of our question, we also collected the following information from each study: the article's ID, type of study (field or laboratory) and the species itself.

### 2.4 Data extraction and effect size

Using the information provided in each study, we calculated the correlation coefficients (*r*) between contest duration and both winners and losers' traits. We then converted these correlation coefficients to an effect size measure (see below). Some studies reported correlations between contest duration and a set of different traits of winners and losers (i.e. Prenter et al., 2006). In this case, we only included the correlation regarding the traits that had a significant relationship with contest success according to the study itself. We did this because traits that determine contest success were the best ones to determine the RHP of each individual (Vieira & Peixoto, 2013). Otherwise, we would be including information regarding individual traits unrelated to RHP and inflating the unexplained variance in our analyses.

Not all selected studies provided a correlation coefficient (*r*), but, when raw data were available, we calculated the correlation coefficient (*cor*() function) using R software (R Core Team, 2021). If the study did not provide the correlation coefficient (*r*) or the raw data, we extracted the regression statistics (such as *t* or *F* values) and used the ‘compute.es’ package in R software (R Core Team, 2021) to calculate the correlation coefficient (*r*). Finally, if we were unable to find any of this information, but the figures contained meaningful information about the raw data, we used the WebPlotDigitizer software (Rohatgi, 2021) to estimate the original data and then calculated the correlation coefficient (R Core Team, 2021). If none of the data above was present, the study was not included.

To obtain the effect size used in our analyses, we transformed the correlation coefficient (*r*) into Fisher's *Z* using the package *compute.es* in the R software (R Core Team, 2021). We performed this transformation because Fisher's *Z* is not bounded between −1 and 1 and is, therefore, more suitable for meta‐analysis (Koricheva et al., 2013). We maintained the original direction, positive or negative, for the correlation between contest duration and winners' and losers' traits following transformation to Fisher's *Z*. Therefore, the transformed value would maintain the same meaning as the original correlation.

We used the existing theoretical models of animal assessment strategies as a guideline to determine if the species adopted self or mutual assessment (Taylor & Elwood, 2003). If the species followed the predictions of E‐WOA, WOA and CAM, we would consider that it adopted self‐assessment. If the species adopted SAM, we would consider it adopted mutual assessment. Positive correlations for losers and non‐significant correlations for winners provide support for E‐WOA and WOA, while positive correlations for losers and negative correlations for winners provide support for CAM and SAM (Taylor & Elwood, 2003). SAM can be distinguished from CAM when there is between‐phase escalation because SAM is the only model that predicts escalation between phases (i.e. a consistent pattern of between‐phase escalation among species), while CAM predicts both between phase escalation and de‐escalation. To determine whether a species exhibited between‐phase escalation, we looked for evidence in the paper's contest description of distinct phases (treated as a specific behaviour, or more rarely, as a group of specific behaviours that changed among themselves during the contest). Then, we checked whether the described phases followed a trajectory from low‐cost behaviours to high‐cost behaviours and whether high‐cost behaviours were preferentially adopted when individuals presented similar values of the traits that indicated RHP. To distinguish between low‐cost behaviour to high‐cost behaviour if it was not already described in the study, we considered that the behaviour that did not involve physical contact were low cost, behaviours that involve physical contact imposed higher cost than the former and the behaviour involving weapons represented the highest cost possible. Some might argue that the model's presence of weapons itself does not represent the highest cost, since some weapons might serve as the individual's RHP signal (Geist, 1966). However, we looked through the contest descriptions to make sure that the weapons were being physically used during the contests either to push, grab or inflict injury. contests as a self‐assessment strategy or a mutual assessment strategy.

### 2.5 Determining contest costs

Estimating the contest costs would ideally involve changes in longevity and reproduction experienced by males during each contest. Since such information is unavailable for most species, we opted to use behavioural and morphological information that is correlated to the potential energetic and injury costs paid by rivals during contests. We did this because, in species in which contests require more energy and/or are highly injurious, we assumed that males would decrease survivorship each time they fought. To perform such a classification, we categorized the contests of each species according to the following three alternatives: (i) the absence of physical contact, (ii) the occurrence of physical contact without the use of weapons (i.e. specialized structures that promote a physical advantage over the adversary) and (iii) the occurrence of physical contact with the use of weapons during the contest. We considered high costs the ones that involved the use of weapons since weapons can be used to pierce, squeeze and impact the rival, and therefore, increase the chances of injuries occurring (Palaoro & Peixoto, 2022). We classified the other two possibilities, contests with physical contact in species without weapons or contests without physical contact, as low cost contests since the chances of injuries are much smaller, with the decision to flee being determined mainly by energy expenditure (Briffa & Sneddon, 2007). Therefore, contests were placed in one of two cost categories: high and low‐cost.

### 2.6 Meta‐analytical model

**FIGURE 1:**
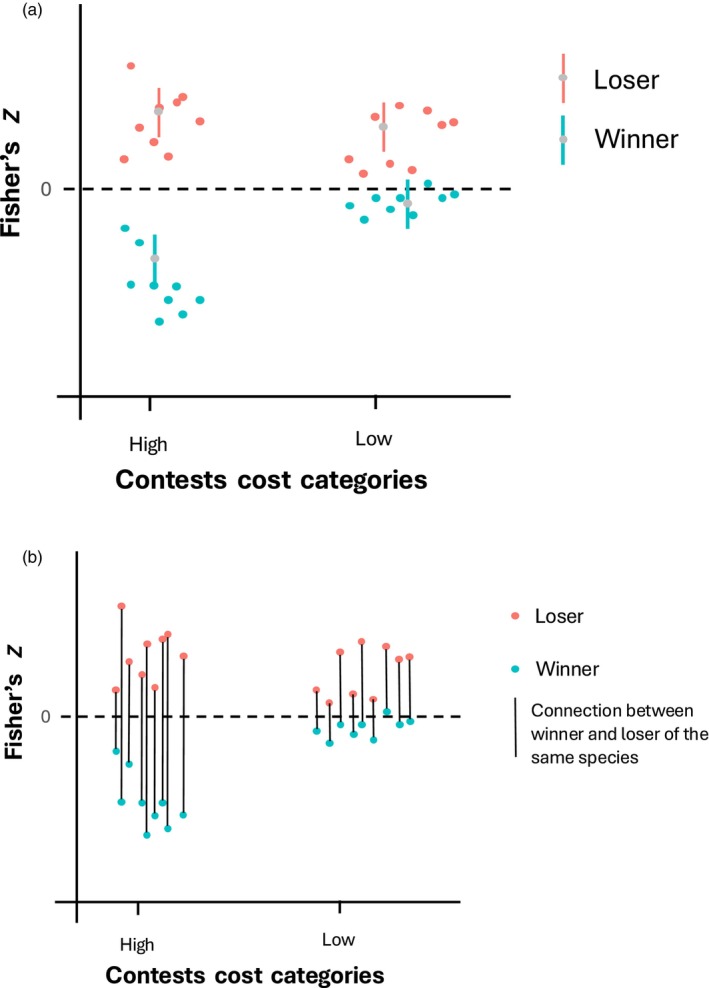
Figure with the predictions of our hypothesis. This shows the expected relationship between contest cost categories and the mean Fisher's *Z* values (along with their 95% confidence intervals). (a) The points represent Fisher's *Z* values for both cost categories. The red points are the loser's values (expected to represent a positive relationship between individual trait and contest duration under both self and mutual assessment), and the blue points are the winner's values (expected to represent a non‐significant relationship between individual trait and contest duration under self‐assessment and a negative relationship between individual trait and contest duration under mutual assessment). The grey point is the mean value, and the bar is the confidence interval. (b) This figure has the same loser's and winner's points, represented by the red and blue, respectively. The black lines are connecting the pair of winner and loser of the same species. This is to make it clearer which strategy each species is presenting.

Our hypothesis states that mutual assessment should be favoured in species that show more costly contests. For this reason, we used a meta‐analytical model to test the association between the two categories of contest costs and the relationship between contest duration and characteristics associated with contest outcome. We obtained mean Fisher's *Z* values that represented correlations between contest duration and individual traits for winners and losers. In species that show more costly contests, we expected that they would preferentially adopt mutual assessment, thus, we expected winners to present, on average, a negative Fisher's *Z* value while losers should show a mean positive Fisher's *Z*. For species with low‐cost contests, where we expected self‐assessment, winners should present a mean Fisher's *Z* similar to zero, while losers should present a mean positive Fisher's *Z* (Figure 1). In addition, in species with high costly contests, between‐phase escalation should prevail.

To test our predictions, we constructed a multi‐level meta‐analytical model (Koricheva et al., 2013). Fisher's *Z* was treated as the response variable, with the individual's status (winner or loser), contest cost (high or low) and their interaction as moderator variables. We added the inverse of variance of Fisher's *Z* as weight in the analysis because studies in which the result presented less variance should be more reliable. We also added species, article ID, type of study (field or laboratory) and sample ID as random variables. We used species ID to control for multiple effect sizes for the same species and article ID to account for any bias arising from multiple effect sizes, such as the information of winners and losers, coming from the same study. We included study type, field or in laboratory, because field studies may present results more similar between themselves when compared to studies performed in laboratory conditions (Pinto & Peixoto, 2019). We also added a phylogenetic covariance matrix to account for phylogenetic effects since more closely related species are expected to present similar behaviours in comparison to distantly related species. Finally, we added the sample ID as a random variable to account for an observation‐level variation (Bolker et al., 2009).

To construct the phylogenetic covariance matrix, we used the *rotl* package (Michonneau et al., 2016) to build a topological phylogenetic tree based on the Open Tree Taxonomy project's online repository (https://tree.opentreeoflife.org). However, some genera in our database were synonymized. Therefore, we changed to the new genus name to obtain the corresponding information in the Open Tree Taxonomy database. After obtaining the topological tree as an ultrametric tree, we used it to build the phylogenetic matrix for the model using the *ape* package (Paradis & Schliep, 2019).

### 2.7 Risk bias across studies

To evaluate publication bias, we conducted a modification of the Egger's test (Egger et al., 1997; Nakagawa et al., 2022). This model entails regressing the Fisher's *Z* values against their respective standard deviation, but using the same random structure adopted in the main analysis (and the species, article ID and type of study as random variables). A significant slope in this model indicates the existence of publication bias (Nakagawa et al., 2022). We also used I^2^ to estimate the heterogeneity among effect sizes. The result is given from 0 to a 100% and indicates the variability in estimates of effect sizes above the variability expected from sampling errors (Higgins et al., 2003; Higgins & Thompson, 2002).

We conducted all analyses in R software (R Core Team, 2021). We used the following packages: *metafor* package to construct and test the meta‐analytical model and the modified Egger's test, *rotl* and *ape* packages to construct the phylogenetic tree with the species in our dataset (using Tree of Life Project, http://tolweb.org/tree/) and *orchaRd* package to calculate heterogeneity across all levels (*I* ^2^).

## 3 RESULTS

The database provided by Pinto et al. (2019) contained 174 effect sizes across 36 species that included all traits measured by the original studies. Following removal of the traits that did not influence contest outcome (following our selection criteria), and three additional studies that did not match our criteria, the number of effect sizes was reduced to 72. Our second search yielded 1350 studies. However, after discarding duplicates, 1204 studies passed to the screening title phase following which 49 studies went through assessment for eligibility. These 49 studies yielded eight additional samples from four new species. Thus, our final dataset resulted in 80 effect sizes (40 losers and 40 winners) for 36 species (Figure S1). Overall, 22 species were categorized as high‐cost and 14 as low‐cost contests (Figure 3). While in most species classified as having high‐cost contests, individuals could use weapons during displays, in all of them, rivals used weapons during physical contact phases of the contest.

**FIGURE 2:**
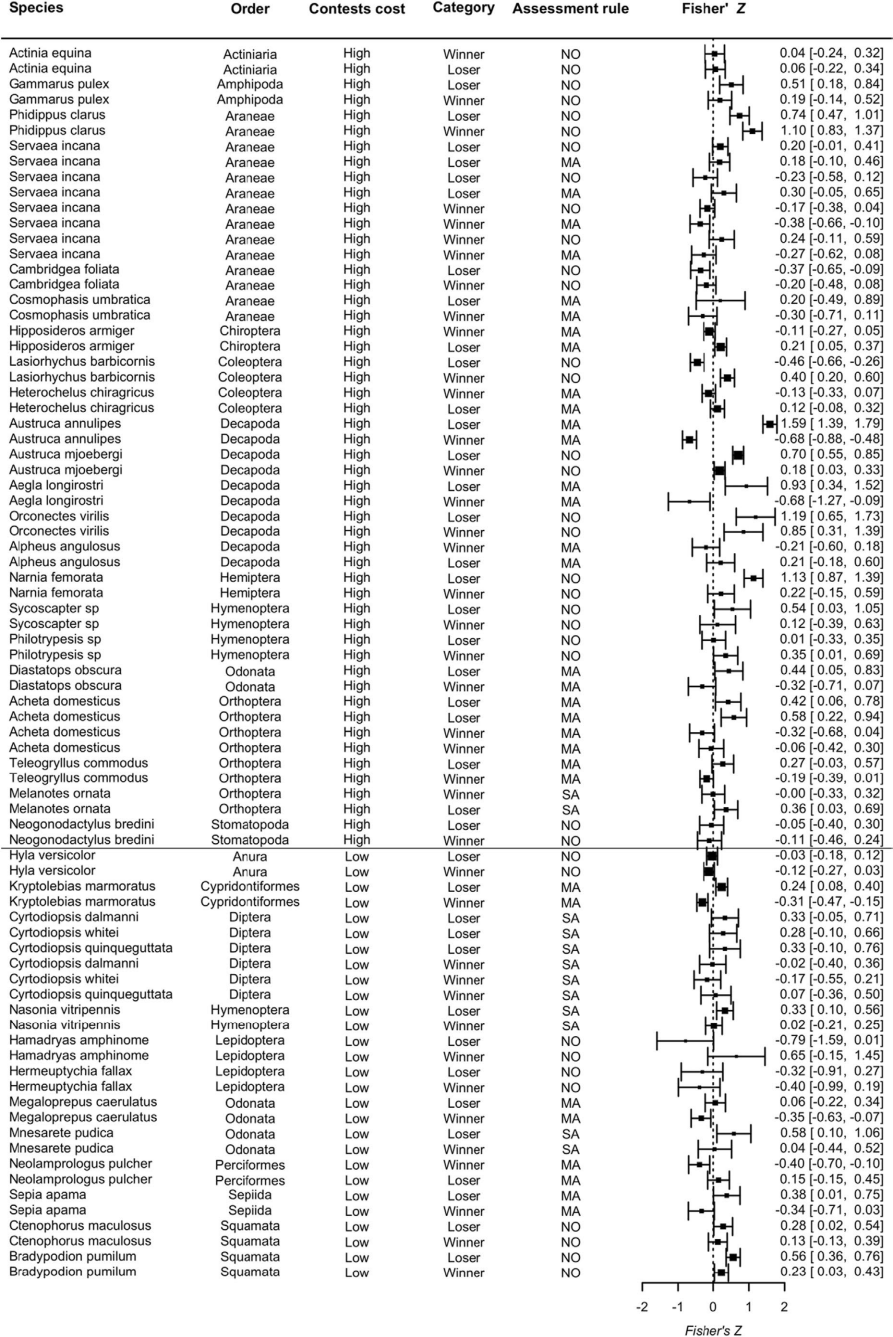
Fisher's *Z* (square) and corresponding confidence intervals (lines) for each trait that indicate RHP in the winner/loser pair of each species. Larger squares denote studies with larger sample sizes. Species are grouped according to their contest cost, in which high‐cost contests involve the use of weapons and physical contact, as described in the Methods section. More details about the species' traits are available in the Table S4.

We found that Fisher's *Z* values reporting the relationship between contest duration and traits linked to RHP changed according to the interaction between contest cost and contest outcome (QM_Contest cost * contest outcome_ = 19.41, df = 3, *p* = 0.002). When contest cost was high, losers' traits had a positive correlation with contest duration, while winners traits had no association with the contest duration (Figure 2a). When contest cost was low, the average Fisher's Z value did not differ from zero for neither winners nor losers. Regarding the relationship between the probability of the contest escalating, we found that 90.9% of the species in the high‐cost category presented escalation, while 71.4% of the low‐cost category presented escalation.

**FIGURE 3:**
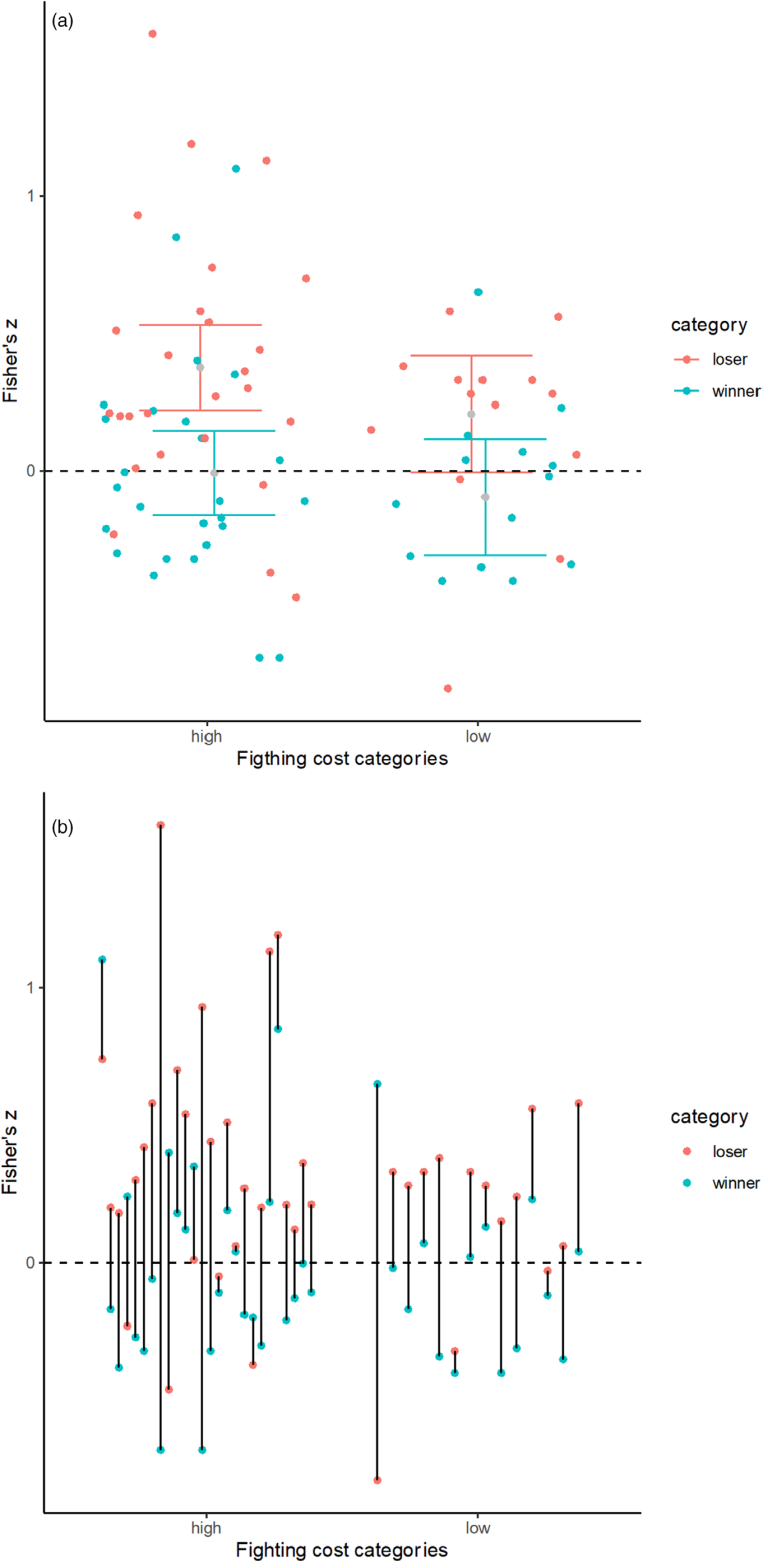
Both graphs show the relationship between contest cost and the assessment model (represented by the Fisher's *Z* values) for each pair of rivals in a species. The dots in red encompass the losers while the dots in blue encompass the winners. (a) Mean Fisher's *Z* values reporting the relationship between contest duration and traits that indicate RHP and the contest costs of different species. Bars represent the 95% confidence intervals. Confidence interval in red encompasses the loser's category while the confidence interval in blue encompasses the winner's category. The means of each category, winners and losers, are represented in grey. (b) Same as ‘a’, but with black lines connecting the winners and losers of each species in each study. Thus, it is possible to assess whether there is evidence for positive, negative or near‐zero results for loser and winners in the same study. If a species presents positive Fisher's *Z* for the loser and negative Fisher's *Z* for the winner, it provides support for mutual assessment. On the other hand, if the species presents a positive Fisher's *Z* for the loser and a close‐to‐zero Fisher's *Z* for the winner, it provides support for self‐assessment. If it does not follow any of the previous predictions, the species does not show support for either self or mutual assessment.

Our model had a high level of heterogeneity (*I* ^2^ = 86.88%); 78.31% of those came from differences between samples (i.e. difference between the effect sizes). Study ID was responsible for 8.56% of the heterogeneity found, while species and phylogeny did not explain heterogeneity (Figure S2). We found no evidence of publication bias (Egger's test: estimate = 0.13, 95% CI = −0.12 to 0.38; *p* = 0.32, Figure S3).

## 4 DISCUSSION

In this study, we investigated whether proxies of contest costs would predict the assessment strategies adopted by different species. We expected that species with high‐cost contests would adopt a mutual assessment strategy; thus, losers would have a positive correlation and winners a negative correlation between contest duration and traits linked to RHP (Figure 1). It is important to note that, although we used quantitative measures of the correlation between individual traits and contest duration, we mainly analysed the average measures obtained in this meta‐analysis, which can provide support for a consistent or non‐consistent pattern among species, since it is the product of the proportion of species in each category showing positive or negative Fisher's *Z* values. Based on this and contrary to our hypothesis, in species with high‐cost contests, contest duration was, on average, positively correlated to the traits that indicate RHP for losers and had no consistent correlation for winners (Figure 2a). This pattern indicates the most species in the high‐cost group follow a self‐assessment strategy, more specifically, the rules postulated by the E‐WOA/WOA models. As for species with low‐cost contests, we expected a self‐assessment pattern, thus, losers would have a mean positive correlation, while winners would present a mean positive but close to zero correlation. However, there was no evidence for a consistent positive or negative correlation for both winners and losers of this category (Figure 2a), showing that assessment strategies among species with low‐cost contests are more variable than assessment strategies adopted by species in the high‐cost group. One could argue that there was also support for self‐assessment in species with low‐cost contests, since the 95% CI associated with the mean Fisher's Z value for losers in this category marginally superimposed zero, while for winners, Fisher's Z greatly encompassed zero (Figure 2a). However, by analysing the pairs of winners and losers for this category (Figure 2b), it is clear that five species appear to adopt self‐assessment, five species appear to adopt mutual assessment, and the remaining four do not follow the predictions of any assessment model. Therefore, based on Figure 2b, it is evident that there is no consistent pattern of assessment strategies for species in the group showing low‐cost contests.

It is surprising that the WOA model prevailed among species in the high‐cost category because it indicates that contests involving physical contact continue until a cost threshold is reached regardless of the opponent's RHP. Although WOA was originally suggested for species in which individuals are unable to cause damage, empirical studies found evidence that WOA can explain assessment strategies in species in which individuals engage in physical contact during contests (Pinto et al., 2019). In fact, for some species in which assessment strategies were evaluated separately on each contest phase, individuals were found to change from mutual assessment strategies during non‐contact phases to self‐assessment during contact phases with weapon employment (Hsu et al., 2008; Lobregat et al., 2019). The adoption of WOA even when individuals enter contests with physical contact may occur, for example, if individuals are cognitively unable to gather information of their rivals during contest phases that incur physical contact (Elwood & Arnott, 2012). Another possible reason for the prevalence of WOA among species with highly costly contests is that there could be a correlation between the presence of weapons and the ability to endure the costs imposed by weapons. If that is the case, animals with bigger weapons would also be better at resisting the costs inflicted by these same weapons. For example, in fiddler crabs, the force of the claw used in contests was positively correlated to the strength of the claw's cuticle that is usually where the rivals grasp each other during contests (e.g. Dennenmoser & Christy, 2013; Swanson et al., 2013). Alternatively, perhaps the individuals of the species with high contest costs are cognitively unable to gather information of their rivals (Elwood & Arnott, 2012).

Another explanation for the prevalence of self‐assessment among species with high‐cost contests may be related to resource value. Investment in large weapons is expected to be favoured in species in which the resource is important (if not crucial) to increase the individual's fitness. In such circumstances, the loser of a species with weapons will incur a stronger reproductive loss than losers in species without weapons. Conversely, a winner in a species with weaponry will incur a higher reproductive advantage than the winners in species without weaponry. In this sense, if the benefits of winning are very high as well as the costs of losing, strategies involving very costly contests may evolve (Enquist & Leimar, 1990). Although suitable comparisons of the lifetime reproductive success of winners and losers in species with distinct assessment strategies are lacking (Maciel et al., 2023), indirect evidence indicates that the resource value may affect the evolution of contest behaviours. For example, in some non‐pollinating fig wasps, males engage in contests for access to females inside figs. Loser males are rarely able to reproduce, and it is common that males use their enlarged mandibles during contests and eventually kill their rivals indicating that males do not quit from contests even when facing stronger opponents (e.g. Bean & Cook, 2001). On the other hand, in damselfly species in which males engage in contests for access to mating sites, loser males can find alternative territories to copulate or adopt alternative mating tactics (Raihani et al., 2008). In these species, individuals do not possess weapons and, in fact, some of them are able to evaluate their opponents during contests (Guillermo‐Ferreira et al., 2015). Therefore, considering the costly nature of contests in species with weapons and our results, we posit that the decision rule may not be influenced by the contest cost itself. Rather, it is the disparity in reproductive success that should be the key factor of the evolution of the assessment strategies.

For species with low‐cost contests, the absence of a consistent pattern for the assessment strategies indicates that the costs that may be acquired during the contest do not seem to drive the evolution of the assessment strategies. By analysing the relationship between contest duration and traits that indicate RHP for species individually, some species provide evidence for self‐assessment under WOA, others for CAM or SAM, while a third group of species does not provide evidence for any specific assessment model (Figure 2b). While species in the high‐cost contests may be susceptible to a strong selective pressure on winning chances because of the reproductive disparity between winners and losers, it may be that this type of selection on species with low‐cost contests is relaxed. If there is a low difference in reproductive success between winners and losers in the low contest cost category, we could expect a relaxation of the selective pressure on the type of decision rule. Relaxed selective pressures tend to allow an increased trait variation during evolution (Lahti et al., 2009). This process of relaxed pressure applied to the species with low‐cost contests would result in an absence of a consistent pattern of assessment strategies.

Based on the patterns we found for the high‐ and low contest cost categories (Figure 2), we conclude that contest cost does not seem to be associated with the evolutionary pathway of the assessment strategies adopted during agonistic interactions. We suggest that, instead of being a driving factor, contest costs may be a result of the selective pressure associated with relative benefits in reproductive success obtained by winners and losers. When the difference in reproductive success between winners and losers is high, self‐assessment strategies that are associated with high investment in contests and with the evolution of weapons may be favoured. On the other hand, when the differences in reproductive success between winners and losers are smaller, selection on the assessment strategies is relaxed and different strategies may evolve. This is an important step in the understanding of the evolution of assessment strategies across species since differences in the costs of contests among them may be a byproduct of selection on contest strategies rather than a cause.

## AUTHOR CONTRIBUTIONS

Paulo Enrique Cardoso Peixoto designed the study. Clara Massote collected the data and, together with Paulo Enrique Cardoso Peixoto, analysed the data and wrote the manuscript. Alexandre V. Palaoro, Gareth Arnott and Domhnall Jennings reviewed the text.

## CONFLICT OF INTEREST STATEMENT

There is no conflict to declare.

## STATEMENT ON INCLUSION

Our work brings together authors of different countries as well as species distributed worldwide. Because all authors had an input into the perspectives that the study brought, we believe that this study brings a unique view onto the matter of the evolution of agonistic behaviours.

## Supporting information


**Figure S1.** PRISMA 2020 flow diagram for updated systematic reviews which included searches of databases and registers only.
**Figure S2.** Ultrametric philogenetic tree of the species included in the meta‐analysis.
**Figure S3.** Funnel graph with the regression of the residual values with the standart error.
**Table S4.** All species used in this study and the following information: species name, order, reference, the RHP morphological trait that determine the contest outcome in each study, contests charateristics (wheather the species had weaponry, the use of contact in the contest and wheather the contest presented escalonation), and, finally, the contest cost category.

## Data Availability

Data available from the Open Science Framework Repository: https://doi.org/10.17605/OSF.IO/UQXK8.
