# Supplementary material for: Do lifetime contest costs affect the evolution of assessment strategies? A meta‐analysis
Source: J Anim Ecol. 2025 May 26;94(7):1335–45. doi: 10.1111/1365-2656.70058 (PMC12214450; doi:10.1111/1365-2656.70058)
Supplement: Supplementary file 1 — Figure S1. PRISMA 2020 flow diagram for updated systematic reviews which included searches of databases and registers only. Figure S2. Ultrametric philogenetic tree of the species included in the meta‐analysis. Figure S3. Funnel graph with the regression of the residual values with the standart error. Table S4. All species used in this study and the following information: species name, order, reference, the RHP morphological trait that determine the contest outcome in each study, contests charateristics (wheather the species had weaponry, the use of contact in the contest and wheather the contest presented escalonation), and, finally, the contest cost category. [file JANE-94-1335-s001.docx]

**Previous studies**

**Identification of new studies via databases and registers**

Studies included in previous version of review (n =34)

Reports of studies included in previous version of review (n =0)

Records identified from*:

Databases (n =2)

Registers (n =1350)

Records removed *before screening*:

Duplicate records removed

(n = 146)

Records marked as ineligible by automation tools (n =0)

Records removed for other reasons (n =0)

**Identification**

Total studies included in review

(n =31)

Reports of total included studies

(n =35)

Reports assessed for eligibility

(n =49)

Reports sought for retrieval

(n =0)

Records screened

(n =1204)

Records excluded**

(n =1155)

Reports not retrieved

(n =0)

**Screening**

Reports excluded:

Did not report variables (n =23)

Theoretic paper (n=5)

Not a natural contest (n =4)

Manipulated RHP (n =3)

Non-random contest (n=4)

Did not separate winners from losers (n=1)

Different study area (n=1)

Interspecific contests (n=1)

Non dyad contest (n=1)

We were not able to convert variable (n=1)

New studies included in review

(n=4)

Reports of new included studies

(n =0)

**Included**

*Consider, if feasible to do so, reporting the number of records identified from each database or register searched (rather than the total number across all databases/registers).

**If automation tools were used, indicate how many records were excluded by a human and how many were excluded by automation tools.

**Fig.1s** PRISMA 2020 flow diagram for updated systematic reviews which included searches of databases and registers only


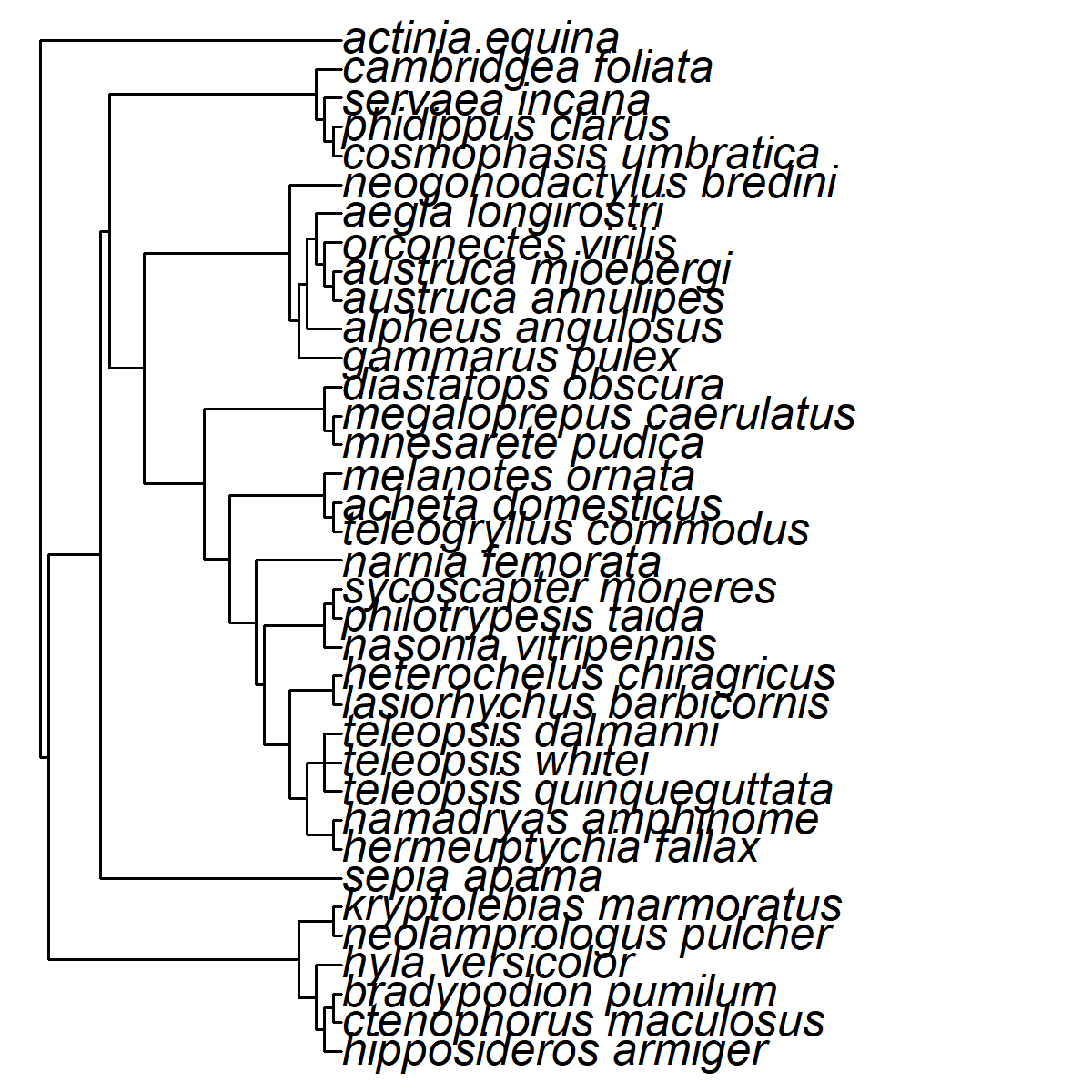


**Fig.2s**. Ultrametric philogenetic tree of the species included in the meta-analysis.


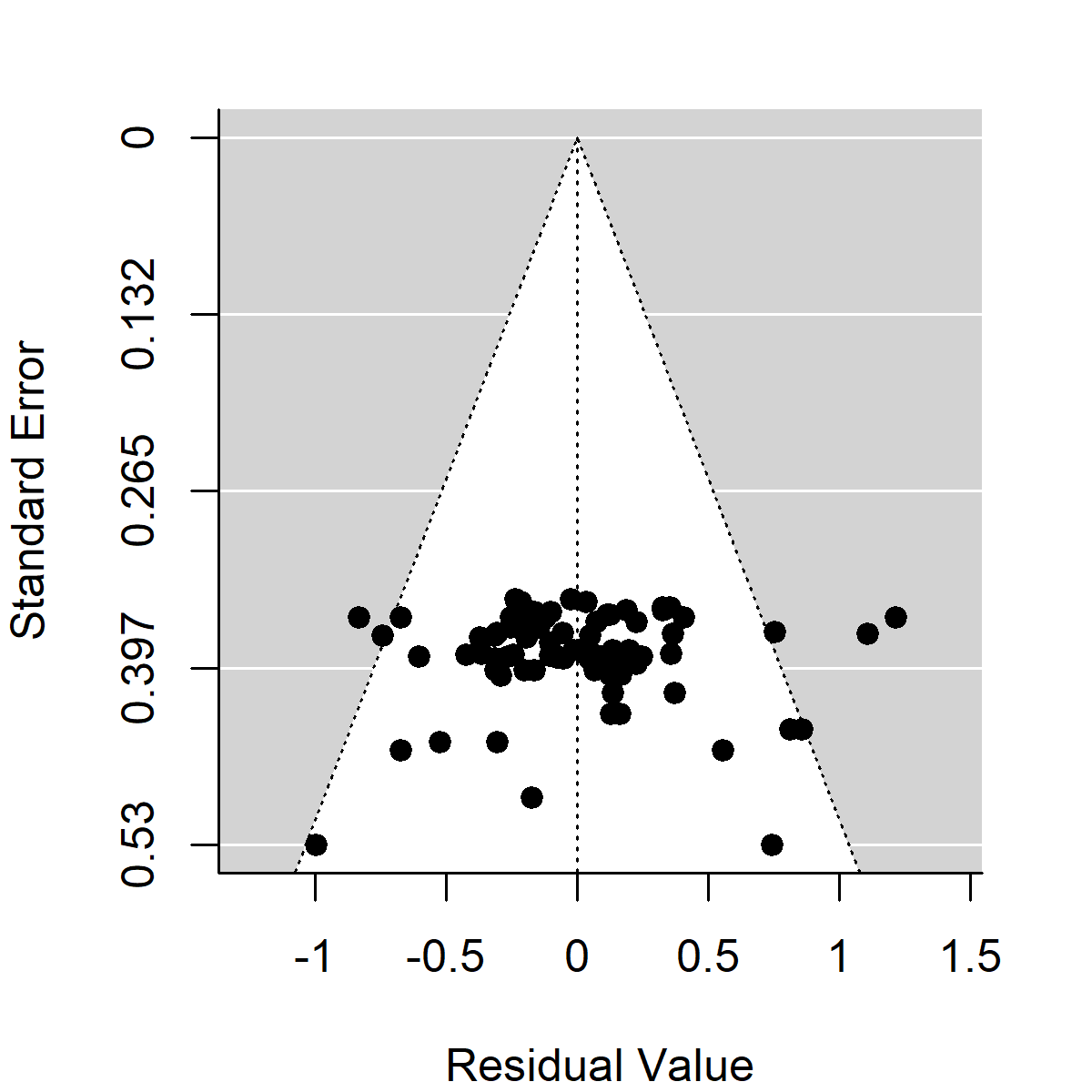


**Fig. 3s**. Funnel graph with the regression of the residual values with the standart error.

| Species | Order | Citation | Year | Atribute | Weapon | Contact | Escalation | Cost caterogory |
| --- | --- | --- | --- | --- | --- | --- | --- | --- |
| *Phidippus clarus* | Araneae | Elias et.al. | 2008 | Vibrations | Yes | Yes | Yes | High |
| *Servaea incana* | Araneae | McGinley et.al. | 2015 | Body size | Yes | Yes | Yes | High |
| *Acheta domesticus* | Orthoptera | Briffa | 2008 | Weight | Yes | Yes | Yes | High |
| *Austruca annulipes* | Decapoda | Bolton et.al. | 2013 | Carapace | Yes | Yes | Yes | High |
| *Hamadryas amphiNome* | Lepidoptera | Lourenço | 2015 | Body mass | No | No | Yes | Low |
| *Cyrtodiopsis dalmanni* | Diptera | Brandt | 2009 | Eye span | No | No | No | Low |
| *Cyrtodiopsis whitei* | Diptera | Brandt | 2009 | Eye span | No | No | No | Low |
| *Cyrtodiopsis quinqueguttata* | Diptera | Brandt | 2009 | Eye span | No | No | No | Low |
| *Lasiorhychus barbicornis* | Coleoptera | Painting & Holwell | 2014 | body length | Yes | Yes | Yes | High |
| *Austruca mjoebergi* | Decapoda | Morrell et.al. | 2005 | claw size | Yes | Yes | Yes | High |
| *Sycoscapter sp* | Hymenoptera | Moore et.al. | 2008 | Mandible length | Yes | Yes | Yes | High |
| *Philotrypesis sp* | Hymenoptera | Moore et.al. | 2008 | Mandible length | Yes | Yes | Yes | High |
| *Aegla longirostri* | Decapoda | Palaoro et.al. | 2014 | Cephalothorax length | Yes | Yes | Yes* | High |
| *Sepia apama* | Sepiida | Schnell et.al. | 2015 | Mantle length | No | Yes | Yes | Low |
| *Hermeuptychia fallax* | Lepidoptera | Peixoto & Benson | 2012 | Body mass | No | No | Yes | Low |
| *Diastatops obscura* | Odonata | Junior & Peixoto | 2013 | Body mass | Yes | Yes | Yes | High |
| *Neogonodactylus bredini* | Stomatopoda | Green & Patek | 2018 | Body mass | Yes | Yes | Yes | High |
| *Nasonia vitripennis* | Hymenoptera | Tsai et.al. | 2014 | Body size | No | Yes | Yes | Low |
| *Ctenophorus maculosus* | Squamata | Mclean & StuartFox | 2014 | Bite force | No | Yes | Yes | Low |
| *Neolamprologus pulcher* | Perciformes | Reddon et.al. | 2011 | Body size | No | Yes | No | Low |
| *Gammarus pulex* | Amphipoda | Prenter et.al. | 2006 | Weight | Yes | Yes | No | High |
| *Kryptolebias marmoratus* | Cypridontiformes | Hsu et.al. | 2008 | Body size | No | Yes | Yes | Low |
| *Actinia equina* | Actiniaria | Rudin & Briffa | 2011 | Dry weight | Yes | Yes | Yes | High |
| *Bradypodion pumilum* | Squamata | StuartFox | 2006 | Performance | No | Yes | Yes | Low |
| *Teleogryllus commodus* | Orthoptera | Reaney et.al. | 2011 | Body size | Yes | Yes | Yes | High |
| *Hyla versiColor* | Anura | Reichert et.al. | 2011 | Body mass | No | Yes | Yes | Low |
| *Cambridgea foliata* | Araneae | Walker | 2018 | Difference in size | Yes | Yes | Yes | High |
| *Megaloprepus caerulatus* | Odonata | Xu & Fincke | 2015 | Band size | No | Yes | Yes | Low |
| *Cosmophasis umbratica* | Araneae | Lim & Li | 2013 | Abdomen total brightness | Yes | Yes | Yes | High |
| *Mnesarete pudica* | Odonata | Guillermo-Ferreira et.al. | 2015 | Relative wing pigmentation | No | No | Yes | Low |
| *Narnia femorata* | Hemiptera | Emberts et.al. | 2018 | Body size | Yes | Yes | Yes | High |
| *Orconectes virilis* | Decapoda | Wofford et.al. | 2015 | Carapace | Yes | Yes | Yes | High |
| *Alpheus angulosus* | Decapoda | Dinh et.al. | 2020 | Carapace lenght | Yes | Yes | Yes | High |
| *Heterochelus chiragricus* | Coleoptera | Rink et.al. | 2019 | Hind femur size | Yes | Yes | No | High |
| *Melanotes ornata* | Orthoptera | Lobregat et.al. | 2020 | Hind femur length | Yes | Yes | Yes | High |
| *Hipposideros armiger* | Chiroptera | Sun et.al. | 2019 | Body mass | Yes | Yes | Yes | High |

**Table 4s.** All species used in this study and the following information: species name, order, reference, the RHP morphological trait that determine the contest outcome in each study, contests charateristics (wheather the species had weaponry, the use of contact in the contest and wheather the contest presented escalonation), and, finally, the contest cost category. To determine de cost category we used contest’s charateristic as mentioned before and classified species in those showing high-, or low-cost contests. We assigned to the high cost category, species in which the contests involves the use of weapons and physical contact and also escalated during the contest. We assigned to the low cost category, species that do not present any of these traits or presents only escalation during the contest.
